# Defining Specific Cell States of MPTP-Induced Parkinson’s Disease by Single-Nucleus RNA Sequencing

**DOI:** 10.3390/ijms231810774

**Published:** 2022-09-15

**Authors:** Yunxia Guo, Junjie Ma, Hao Huang, Jitao Xu, Chao Jiang, Kaiqiang Ye, Ning Chang, Qinyu Ge, Guangzhong Wang, Xiangwei Zhao

**Affiliations:** 1 State Key Laboratory of Bioelectronics, School of Biological Science & Medical Engineering, Southeast University, Nanjing 210096, China; 15150517535@163.com (Y.G.); haohuang@seu.edu.cn (H.H.); jitao_xu1997@163.com (J.X.); njutcm_jc@126.com (C.J.); kaiqiangye1104@163.com (K.Y.); chang_ning_i@163.com (N.C.); geqinyu@seu.edu.cn (Q.G.); 2 Shanghai Institute of Nutrition and Health, University of Chinese Academy of Sciences, Shanghai 200031, China; majunjie2018@sibs.ac.cn

**Keywords:** Parkinson’s disease, 1-methyl-4-phenyl-1,2,3,6-tetrahydropyridine, single-nucleus RNA sequencing, cellular states, cell–cell communications

## Abstract

Parkinson’s disease (PD) is a neurodegenerative disease with an impairment of movement execution that is related to age and genetic and environmental factors. 1-methyl-4-phenyl-1,2,3,6-tetrahydropyridine (MPTP) is a neurotoxin widely used to induce PD models, but the effect of MPTP on the cells and genes of PD has not been fully elucidated. By single-nucleus RNA sequencing, we uncovered the PD-specific cells and revealed the changes in their cellular states, including astrocytosis and endothelial cells’ absence, as well as a cluster of medium spiny neuron cells unique to PD. Furthermore, trajectory analysis of astrocyte and endothelial cell populations predicted candidate target gene sets that might be associated with PD. Notably, the detailed regulatory roles of astrocyte-specific transcription factors Dbx2 and Sox13 in PD were revealed in our work. Finally, we characterized the cell–cell communications of PD-specific cells and found that the overall communication strength was enhanced in PD compared with a matched control, especially the signaling pathways of NRXN and NEGR. Our work provides an overview of the changes in cellular states of the MPTP-induced mouse brain.

## 1. Introduction

Parkinson’s disease (PD), a prevalent neurodegenerative disease, is predominantly characterized by motor disorders, followed by non-motor symptoms including cognition impairments, autonomic dysfunction and hyposmia [1]. PD mainly affects the elderly, accounting for a prevalence of 1.7% in the population aged over 65 and the number of PD patients increases with aging, which causes serious health problems and care costs for the elderly and their families. Currently, PD is universally acknowledged to be caused by neuronal death in substantia nigra [2], the degeneration of dopaminergic neurotransmission and the accumulation of a-synuclein (Lewy bodies) in neuronal cells [3]. However, PD is presently incurable, and the underlying mechanisms behind the neurological degeneration have been the subject of intense study over the last two hundred years.

Single cell/nucleus RNA sequencing (sc/snRNA-seq) technology has emerged as the most powerful instrument for assessing cell-type heterogeneity [4], and this technique has been widely used in neuroscience. To date, the majority of previous sc/snRNA-seq studies on PD have focused on iPSC-derived dopamine neurons [5,6] and mutant mouse (*LRRK2, SNCA*) postmortems brain [7,8,9]. However, most PD cases are sporadic, and up to 15% of PD cases are related to genetic mutations, but various environmental factors can also induce PD-like symptoms. MPTP is a neurotoxin that can cause PD symptoms such as bradykinesia, postural instability, rigidity, cognitive deficits and temporary autonomic disturbances [10]. MPTP can cross the blood–brain barrier (BBB) and be oxidized to 1-methyl-4-phenylpyridinium (MPP^+^) by monoamine oxidase B, and then MPP^+^ is concentrated in the dopaminergic terminals and cell bodies by the dopamine uptake transporter to produce toxicity [11]. This process is often accompanied by astrogliosis and microgliosis and endothelial cell injury [12]. However, all of these results were derived from traditional techniques such as immunohistochemical and positron emission tomogram imaging [12]. In addition, a current study on the MPTP-PD transcriptome is limited to RNA-seq for bulk tissues [13]. Although these studies provide valuable insights into the cellular phenotypic effects of MPTP on the mouse brain, how MPTP affects the cell states at the single cell transcriptional level has yet to be elucidated.

Here, we applied snRNA-seq to investigate complex cellular state changes in the brain tissue of MPTP-PD and matched control (CN) mice. Firstly, we identified PD-specific astrocytes and endothelial cells based on cell proportion, cell density, differential expression genes and transcriptional regulation analysis. Then, the activation states of PD-specific cells were characterized by trajectory reconstruction analysis, and the gene sets that may mediate PD development were discovered. Moreover, another PD-related cell, PD-exclusive D2-medium spiniform neuron (D2-MSN), was identified through the re-clustering of PD-deficient neurons, which might be an independent cellular state caused by MPTP induction. Eventually, we analyzed the changes in the communication relationship between PD-specific cells to explore the effects of MPTP on the communication pattern of these cells. Altogether, our work lays the foundation for elucidating the effect of MPTP on the cellular heterogeneity of brain tissue in PD, and we expect that our study will significantly facilitate future studies in PD mechanisms.

## 2. Results

### 2.1. Single-Nucleus Transcriptome Profiling to Identify Cell Populations

**Figure 1:**
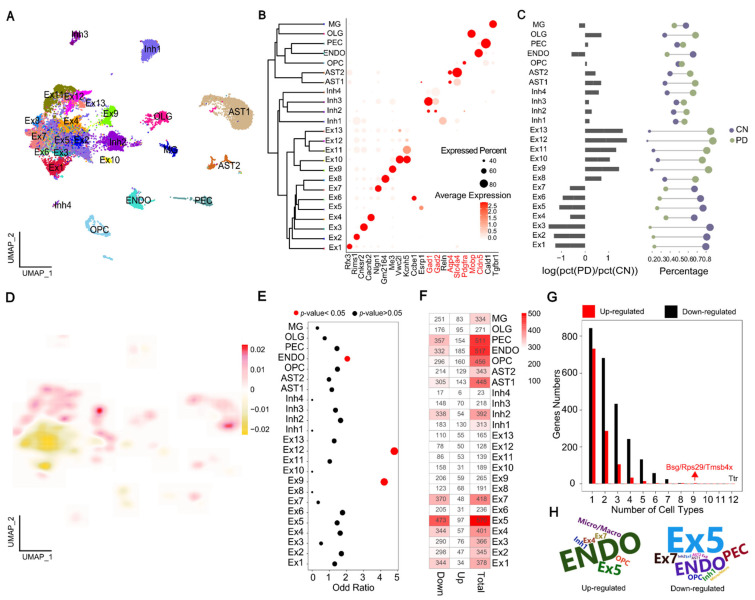
The characterization of cellular diversity in MPTP-PD brain by snRNA-seq. (**A**) Contribution of nuclei from PD or CN to each cell type; colored by cluster. (**B**) Cell representative marker genes. Expression level (color scale) of marker genes across clusters and the percentage of cell expression (dot size). (**C**) The changes in frequency of multiple cell types between PD and CN. Left: log ratio of average fraction in PD vs. CN. Right: proportion of PD and CN profiled cells. The color and dot size represent different samples and the percentage of cells, separately. (**D**) Differential 2D cell density between PD and CN. Red and yellow indicate the high and low density of cells in PD, respectively. (**E**) Using Fisher’s exact test to obtain cell types in which PD-risk gene enrichment. Circle size indicates OR value, and red color highlights enriched cell types with *p*-value < 0.05. (**F**) The number of cell-specific DEGs. (**G**) The number of up- or down-regulated genes per cell type was detected. (**H**) PD-risk DEGs’ detected frequency in each cell type.

To investigate the effects of MPTP on the cellular heterogeneity of the brain in PD, snRNA-seq was performed on the mixed samples of four brain regions from MPTP-PD and CN mice (Figure S1A), which have been shown to be associated with PD in the previous work [13]. After filtering out potential doublets, and poorly sequenced and damaged nuclei, 19,531 high-quality nuclei were kept for downstream integrated analysis (Figure S1B,C). After batch correction, 24 clusters were identified and showed largely similar cellular landscapes in MPTP-PD and CN (Figure 1A and Figure S1D), and the results of cell correlation further confirmed the accuracy of cell classification (Figure S2A). These clusters were manually identified on the basis of the expression of known cell-type-specific markers; eight major cell types were annotated: excitatory neurons (Ex1–13), inhibitory neurons (Inh1–4), astrocytes (AST1–2), microglia (MG), an oligodendrocyte cell (OLG), oligodendrocyte precursor cell (OPC), endothelial cell (ENDO) and pericyte (PEC) (Figure 1B and Figure S2C and Table S1). To investigate the changes in cell-type composition associated with MPTP-PD, three approaches were used. Initially, we examined changes in the composition of each cluster in the context of disease and found several that were overrepresented (Ex8–13, AST, OLG) or underrepresented (Ex1–7, ENDO) in MPTP-PD, and the proportion of other cells was similar to CN (Figure 1C). Subsequently, we compared PD and CN cell density distributions in the UMAP representation and found that the fraction of AST1, Ex4 and Ex9 in MPTP-PD were increased compared to CN (Figure 1D and Figure S2C). In addition, we evaluated whether the expression patterns of PD-associated risk genes were cell-specific. The results showed that PD-risk variants were significantly enriched in Ex9, Ex12 and ENDO cells (*p*-value < 0.05, OR > 1) (Figure 1E). Altogether, these results preliminarily predicted that AST1, ENDO and excitatory neurons might be the cell types with the most obvious effects of MPTP on PD.

### 2.2. Multi-Dimensional Validation of MPTP-PD Specific Cells

To verify our prediction of PD-specific cells, we investigated further from multi-dimensions including differential expression genes (DEGs) and transcriptional regulation analysis. We compared the numbers of cell-type DEGs between PD and CN, and found that about 75% of DEGs were down-regulated in PD, especially in Ex1–7, AST1 and ENDO (Figure 1F). The detected frequency analysis showed that the DEGs had strong specificity in each cell type, and the number of detected down-regulated genes were more than that of up-regulated genes in PD (Figure 1G). The down-regulated gene *Ttr* could be identified in half of cell types, while up-regulated genes *Bsg*, *Rps29* and *Tmsb4x* were detected in nine clusters (Figure 1G), and all of them were dysregulated in ENDO and Ex5 cells (Table S2). With regards to this, Cakar et al. revealed that polyneuropathy can be caused by the accumulation of amyloidogenic Ttr protein in tissues, as in Alzheimer’s disease (AD) and PD [14]. P53 mediates cell defects associated with *Rps29*, and p53 inhibitors were very effective in maintaining motor function in PD mice [15]. Overexpression of *Tmsb4x* in cultured hippocampal neurons can reportedly reduce neurite outgrowth and neuronal development [16]. Notably, a greater number of PD-risk DEGs were obtained in ENDO and Ex5 cells in MPTP-PD (Figure 1H).

**Figure 2:**
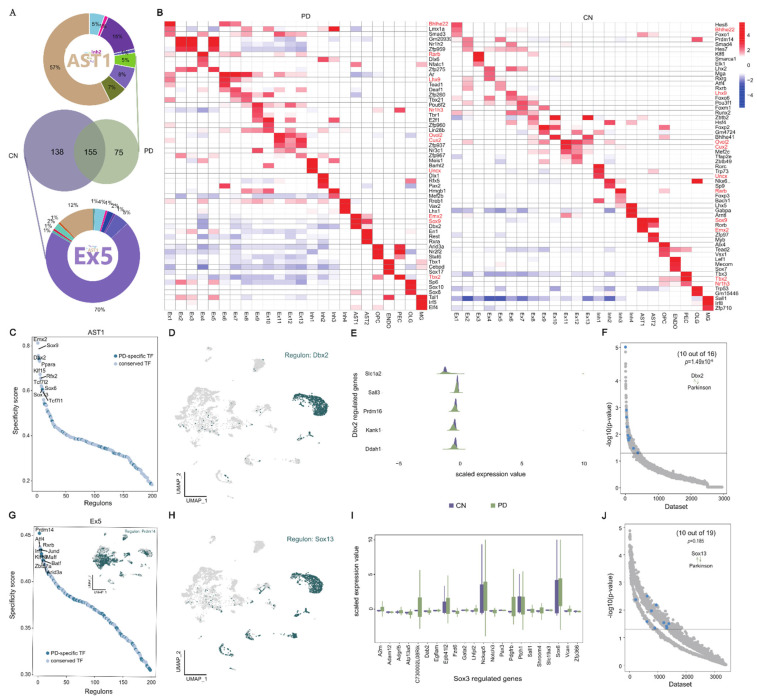
Cell type-specific transcription factors in disease. (**A**) Venn plots of conservative and specific TFs in PD and CN. Middle Venn plot shows conservative and specific TFs detected in PD and CN. The circle percentage diagram with word cloud insert detailed presents that specific TFs of CN and PD were mainly derived from Ex5 and AST1; the colors of circle diagram and word cloud plot correspond. (**B**) Heatmap of top three specific TFs for each cell type in PD (**left**) and CN (**right**). (**C**,**G**) The rank of regulons in AST1 and Ex5 based on regulon specificity score. (**D**,**H**) Binarized regulon activity scores for top regulons Dbx2 and Sox13 on UMAP map (dark green dots). (**E**,**I**) Expression levels of Dbx2 and Sox3 transcription-regulated target genes in AST1. (**F**,**J**) SEEK co-expression result for target genes of top regulons Dbx2 and Sox13 in different public datasets. The x-axis represents different datasets, and the y-axis represents the co-expression significance of target genes in each dataset; AST1-related datasets with significant correlation (*p*-value < 0.01) are highlighted by blue dots.

Transcription factors (TFs) tightly control cell fate in neurodevelopment and have been implicated in neurodegenerative processes [17]. Therefore, we validated MPTP-PD specific cell types from the perspective of transcriptional regulation, and further explored the effect of TFs on disease. We identified 213 and 293 significant TFs in MPTP-PD and CN, respectively, and most of the CN- and PD-specific TFs were contributed from Ex5 and AST1 (Figure 2A). The heatmap of the top 3 specific TFs of each cell type showed the activation status of specific regulatory factors in each cell type, among which only 10 TFs (Bhlhe22, Lhx9, Ovol2, Cux2, Uncx, Rarb, Sox9, Emx2, Tbx2, Nr1h3) were co-activated in the same cell types of PD and CN, but the regulatory intensity was different (Figure 2B). It suggested that alterations in the activation of TFs may drive changes in disease cell states. Subsequently, we focused on 155 overlapped TFs from all clusters between PD and CN and found that 101 (65%) co-regulated conserved TFs have significant similar activated states among all clusters between PD and CN, while the remaining TFs have strong cell-specific activated patterns in PD or CN (Figure S3). It was suggested that the activation of some TFs with cell-type specificity might be revealed by the changes in the intracellular transcriptional regulatory network, thus affecting the development of PD. Finally, the activation status of the other 54 TFs suggested that there was a general homogeneity in the activation or inhibition of TFs in all cell types; only Rarb and Foxp2 were simultaneous activated and inhibited in different cell types, and Maf and Xbp1 were activated or inhibited in almost all neurons of PD, respectively (Figure S4). In conclusion, we systematically revealed candidate trans-regulatory elements in different cell types of MPTP-PD for the first time, especially disease-related AST1.

Taken together, the results of cell proportion, cell density and PD-risk gene enrichment analysis preliminarily predict that AST1, ENDO cells and neuron cells might be associated with PD. Subsequent results of DEGs and transcriptional regulation further verified our hypothesis. For example, the number of down-regulated genes in EX1–7, AST1 and ENDO cells was prominent, the up- and down-regulated PD-risk genes were mainly from Ex5 and ENDO cells, and 57% of PD-specific TFs were from AST1. Therefore, our multi-dimensional methods ultimately focused on PD-specific cell types: AST1, ENDO and PD-deficient neurons, which will be the focus of further research.

### 2.3. Transcriptional Regulation of Disease-Specific Astrocytes

To investigate the specific TFs of AST1 and Ex5 cells and their regulatory roles in PD, we sought to evaluate the cell-specific TFs’ activation states. Dbx2 and Sox13 were identified as the most prominent specific TFs associated with AST1 in MPTP-PD (Figure 2C), and Prdm14 was the top TF that was associated with Ex5 in CN based on the rank of regulon specificity score (Figure 2G). The UMAP plot provided additional support that the activities of Dbx2 and Sox13 were highly specific to AST1 (Figure 2D,H), but Prdm14 was not only activated in Ex5 (Inset of Figure 2G). Subsequently, the genes regulated by Dbx2 and Sox13 were identified by RcisTarget [18], and the expression levels of these genes were investigated in PD and CN, respectively. The genes that were regulated by Dbx2 were under-expressed in PD, while the expression of Sox13-regulated genes were opposite, indicating that Dbx2 and Sox13 may act as a transcriptional inhibitor and activator in MPTP-PD (Figure 2E,I). To further evaluate the accuracy of our findings, we applied SEEK analysis to search for GEO datasets about the co-expression pattern of Dbx2 and Sox13 target genes, then highlighted the work title that co-occurred with the term ‘Parkinson’, and found that these co-expressed genes tend to be associated with PD (Figure 2F,J and Table S3). For example, Slc1a2, Prdm16, Kank1 and Ddah1 were target genes of Dbx2, the high expression of Slc1a2 was found to reduce the risk for PD in a Chinese cohort [19] and the other genes are associated with cognitive function [20], autism spectrum disorder [21] and depression-like behavior [22], respectively. Meanwhile, Sox13 target genes Ptch1, Pdgfrb, Vcan, Lhfpl2 and A2m have been reported to be related to PD. Other regulated genes by Sox13 might be associated to PD syndrome; for example, Nckap5 is considered the most promising candidate for bipolar disorder [23], and Epb41l2 gene is associated with cognitive impairment in the hippocampus induced by anesthesia [24]. Thus far, the study about Dbx2 and Sox13 has focused on neural stem cells [25], and the role of Dbx2 and Sox13 in PD has not been studied.

### 2.4. Trajectory Reconstruction of MTPT-PD-Associated Astrocytes and Endothelial Cells

**Figure 3:**
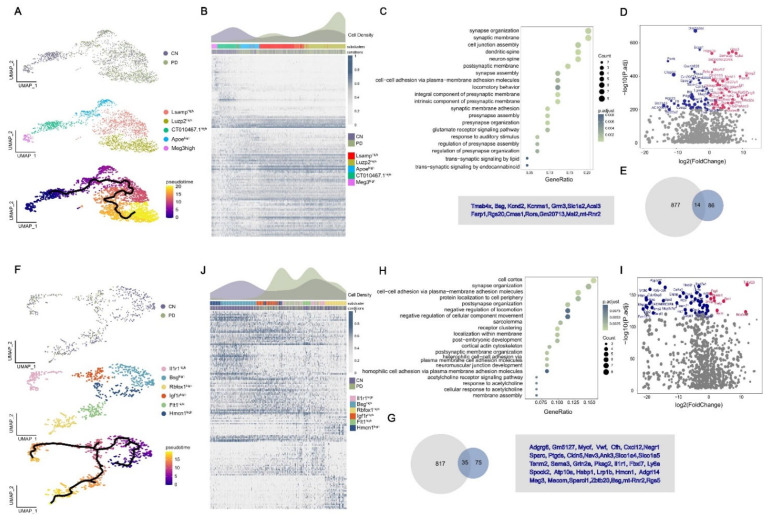
Trajectory reconstruction reveals astrocyte and endothelial differential activation in PD. (**A**,**F**) AST1 and ENDO subclusters labeled with a representative marker gene and trajectory reconstruction and pseudotime representation of subclusters. (**B**,**J**) PD and CN differential cell density distribution along pseudotime. The expression of 100 and 113 genes highly associated with the AST1 and ENDO activation trajectory, respectively. (**C**,**H**) KEGG and GO terms associated with genes of the Luzp2^High^ and Lsamp ^High^ cells in AST1 and Hmcn1^High^, Igf1r^High^, Flt1^High^ in ENDO, respectively. (**D**,**I**) Volcano map of DEGs in PD and CN. The up-regulated genes with red dots, down-regulated genes with blue dots. (**E**,**G**) The overlapped genes between PD-DEGs and the DEGs along the AST1 and ENDO activation trajectory.

To investigate the changes in AST1 and ENDO cell states in MPTP-induced mice, we subclustered these cells and reconstructed their activation trajectories. We identified five AST1 subpopulations characterized by high expression of *Meg3*, *CT010467.1*, *Apoe*, *Lsamp* and *Luzp2* (Figure 3A and  Figure S5A). Subsequently, we reconstructed a cell trajectory structure comprising these major subpopulations using the DDRTree method of Monocle3 [26]. The activation trajectory of AST1 spans from Meg3^High^ cells towards two activation branches, one containing Apoe^High^ cells and the other with clusters highly expressing *Luzp2* and *Lsamp* (Figure 3A). It has been reported that the relative expression level of *Meg3* in PD patients is lower than that in the healthy population [27], while *Apoe* has an impact on the cognitive decline of PD [28]. *Luzp2* is found to be associated with AD [29], schizophrenia [30], intelligence [31] and verbal memory [32], and the level of *Lsamp* is increased in both patients with depression and schizophrenia [33]. Importantly, we observed that these five subclusters were all distributed in AST1 cells of UMAP; in particular, the clusters with high *Luzp2* and *Lsamp* expression were in the cells of increased AST1 in cell density analysis (Figure 1E and Figure S5B). We observed that *Luzp2* and *Lsamp* genes were distributed in the hippocampal based on the results obtained from in situ hybridization of the Allen Brain Atlas (Figure S5C). To further characterize the linked genes of these activated AST1 states in PD, we identified 100 genes whose expression was associated with the activation trajectory, of which 42 and 48 genes were independently highly expressed in CN and PD, respectively (Figure 3B). To identify the potential pathways associated with marker genes of Lsamp^High^ and Luzp2^High^ subclusters, we performed functional annotation using a hypergeometric test based on the Kyoto Encyclopedia of Genes and Genomes (KEGG) database. These two subpopulations were associated with locomotory behavior, the trans-synaptic signaling of endocannabinoid, response to auditory stimulus and glutamate receptor pathways (FDR < 0.05) (Figure 3C). Next, we performed functional annotation for PD of up- and down-regulated genes (Figure 3D and Table S4) in all AST1 subpopulations based on the molecular function of the Gene Ontology (GO) database. The results showed that PD-up-regulated genes were associated with synaptic, dendritic/neuron spine, startle response, synaptic transmission and ion channel regulator activity (FDR < 0.05) (Figure S5D), which were associated with PD in previous studies [34,35,36]. Meanwhile, 14 overlapped genes were obtained between the DEGs and the activation trajectory-associated genes in AST1 (Figure 3E and Table S4). Although none of these genes overlapped with existing PD-risk gene sets, most of them were candidate genes related to autism [37], dyskinesia [38] and schizophrenia [39]. We speculated that these genes might be involved in the development of PD.

**Figure 4:**
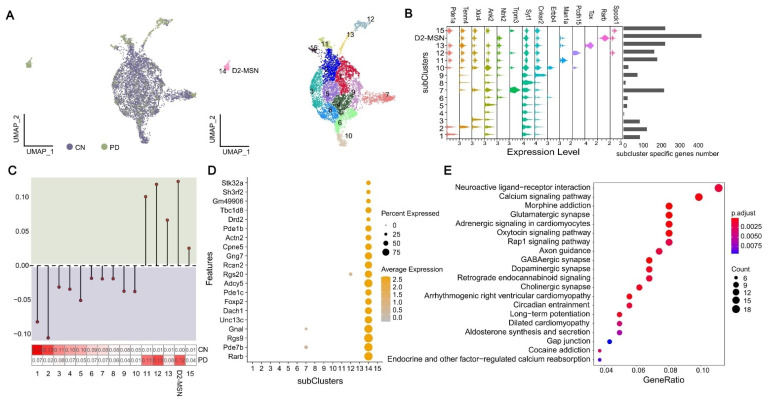
Neuronal states specific to disease models. (**A**) UMAP dimensionality reduction of Ex1–8 from the snRNA-seq analysis. (**B**) Marker gene expression and the number distribution of cluster-specific higher expressed genes. (**C**) The proportion of CN and PD in 15 subclusters. Top: the lollipop of CN vs. PD, y > 0 means that the proportion of cells in PD is greater than CN; bottom: heatmap of the cells’ proportion in PD and CN. (**D**) Bubble chart of all marker gene expression in subcluster 14. (**E**) KEGG terms associated with genes of subcluster 14.

Following the same analytical approach mentioned above, we identified six ENDO subclusters characterized by the high expression of *Hmcn1*, *Bsg*, *lgf1r*, *ll1r1*, *Flt1* and *Rbfox1* (Figure 3F and Figure S6A,B), and recovered their activation trajectory. The results implied an ENDO activation transited from Hmcn1^High^ to Rbfox1^High^ and ll1r1^High^ subclusters (Figure 3F). We observed that ENDO cells were generally absent in PD, but the cells of Bsg^High^ were most severely absent, showing almost completely deletion (Figure S6C). The *Bsg* gene is specifically expressed in ENDO cells of the brain, and it has been reported that *Bsg* knockout mice exhibited deficits in learning and memory [40]. Indeed, ENDO cells in PD were highly enriched at the two activation branches of their trajectory (Rbfox1^High^ and ll1r1^High^) compared to CN (Figure 3J and Figure S6D). *Rbfox1* is one of the risk genes that are common to PD and various psychiatric disorders [41]. *Ll1r1* can be regulated by miRNAs that have been implicated as the potential regulators of alcohol-related neuroinflammation, inducing brain injury and neurodegeneration [42]. Subsequently, we performed functional annotation for Hmcn1^High^, Igf1r^High^ and Flt1^High^ subcluster marker genes based on the GO database; the results showed that they were highly functionally related to the negative regulation of locomotion and cellular component movement, cell adhesion and the acetylcholine receptors (FDR < 0.05) (Figure 3H). The acetylcholine receptors may be stimulated by endogenous agonists such as acetylcholine, or exogenous chemicals such as nicotine, to activate physiologic angiogenesis or pathologic angiogenesis [43]. Next, we identified 35 overlapped genes between DEGs and the activation locus genes of ENDO cells (Figure 4I,G and Table S4). About half of the overlapped genes have been found to be related to PD in previous studies, and the other genes are related to neuropsychiatric diseases (*Zbtb20*, *Nav3*), tissue aging (*Myof*), impaired memory (*Atp10a*) and the blood–brain barrier (*Slco1a4*, *Cldn5*, *Ly6a*) [44,45,46].

Since a previous study indicated that OLG and OPC were related to PD [9], we also performed trajectory analysis for OLG and OPC cells. Compared with AST1 and ENDO, the trajectory and cell density distributions analysis of OLG and OPC showed more similar states, and the heatmap of the trajectory-dependent genes’ density also showed the similar pattern (Figure S7).

### 2.5. Characterization of Transcriptomic State of Neuron Cells

In order to explore the effect of MPTP on neurons in the process of inducing PD, we attempted to decipher the identity of the neurons that were absent in PD. We separated all excitatory neuron cells into two major groups, Ex1–8 and Ex9–13, according to gene expression similarity (Figure 1B, hierarchical cluster diagram). Almost all subclusters in Ex1–8 showed the absence of the PD cell except for Ex8 (Figure 1C). We re-clustered Ex1–8 to distinguish 15 subclusters (Figure 4A) and surprisedly found that subcluster 14, consisting of 206 cells, had no continuity with other subclusters and was only concentrated in PD (Figure 4A,C). Moreover, subcluster 14 was derived from cells with increased Ex4 in cell density distribution analysis (Figure S8A). The number distribution of cluster-specific higher expressed genes showed that subcluster 14 had the most marker genes (Figure 4B), and the top four highly expressed genes of subcluster 14 could be verified in the striatum of the Allen Brain Atlas (Figure S8C). Among them, the mutations in *Rarb* could cause intellectual disability with progressive motor impairment [47], and *Pde7b* plays an important role in schizophrenia [48] and dopaminergic cell death [49]. *Rgs9* is a potent modulator of G-protein-coupled receptor function in striatum [50], and dopamine receptors are associated with distinct G-proteins [51]. The mutations in the *Gnal* could cause primary torsion dystonia [52]. In addition, some other marker genes of subcluster 14 have been extensively studied. The recent literature report indicated *Dach1* expression in the human striatum MSN [53]. *Adcy5* mutations have been associated with substantia nigra damage, and white and gray matter changes in striatal cortical pathways [54]. Expression levels of *Rcan2* were responsive to external stressors such as reactive oxygen species, Ca^2+^, amyloid beta and hormonal changes and are up-regulated in degenerative neuropathy [55]. *Gng7* is the abnormal protein of dopaminergic signaling [56]. *CPne5* is the circadian rhythm-related proteins, and circadian rhythm has a direct or indirect effect on the neurodegenerative processes [57], and more importantly, the gene is involved in PD-induced toxins such as paraquat [58]. In accordance with the expression characteristics of these genes (Figure 4D), we defined subcluster 14 as the PD-exclusive D2-MSN that was located in the striatum. Finally, we performed KEGG and GO analysis on the marker genes of D2-MSN and found that all terms are significantly associated with neuronal synapse (e.g., dopaminergic, glutamatergic, cholinergic, GABAergic), the ligand-receptor interaction pathway, ion channel and other related functions (FDR < 0.05) (Figure 4E and Figure S8D). A study has shown that a-synuclein can induce the dysregulation of miRNAs, which target the neuroactive ligand–receptor interaction pathway [59]. In conclusion, combined with functional analysis and the literature review of marker genes obtained from subcluster 14, we speculated that this subcluster was in an independent cell state during MPTP induction.

### 2.6. Analysis of Cell–Cell Communication in MPTP-PD Specific Cells

**Figure 5:**
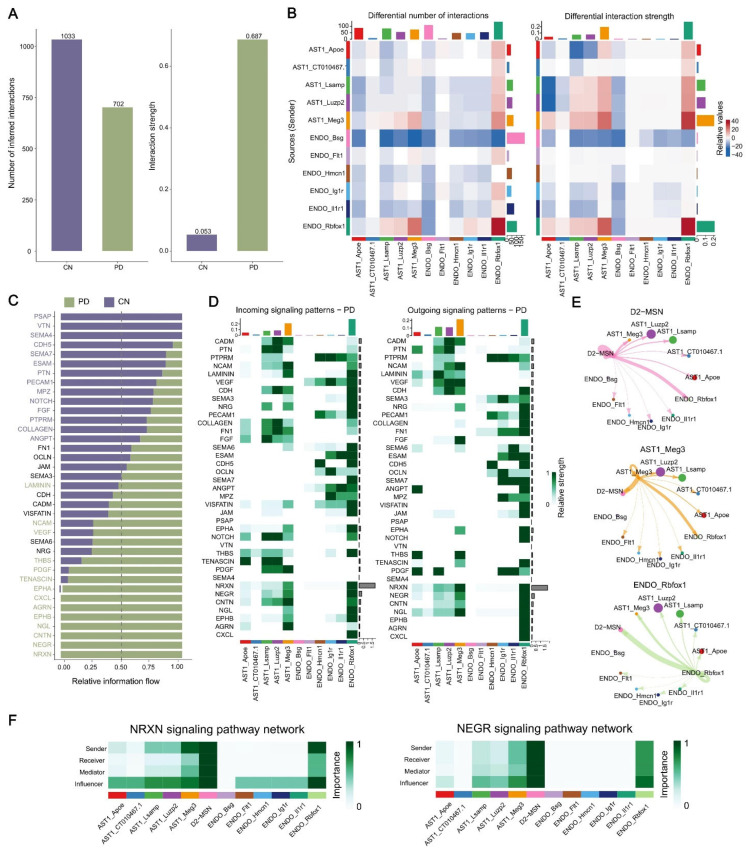
Characterization of cell communications among PD-specific cells. (**A**) The number and strength of AST1 and ENDO intercellular ligand–receptor interactions in PD and CN. (**B**) Heatmaps of the interaction quantity (left) and strength (right) between AST1 and ENDO subpopulations in PD and CN. (**C**) Identification and visualization of conserved and specific signaling pathways. (**D**) Heatmaps of the outgoing and incoming signaling patterns of AST1 and ENDO subclusters in PD. (**E**) Circle plots show and compare cell–cell communication alterations among PD-specific cells. (**F**) Heatmaps of the NRXN and NEGR signaling networks displaying relative importance of each cell group ranked.

Integrating pathways and functions of all PD-specific cells suggested that MPTP was likely to alter cell–cell communication. For example, the top enriched terms in D2-MSN included neuroactive ligand–receptor interaction and the calcium signaling pathway, synaptic membrane and cell junction assembly in AST1, the cell–cell junction in ENDO and so on. To further explore the interactions between PD-specific cells, we applied CellChat to infer intercellular communication networks. The changes in cell communication analysis require the same cell population composition between two datasets. Thus, we first used AST1 and ENDO cells between PD and CN. We found that the global number of ligand–receptor (L-R) pairs was decreased in PD, while the interaction strength was enhanced in PD compared to CN (Figure 5A,B). Interestingly, although ENDO_Rbfox1^High^ and AST1_Meg3^High^ cells were reduced in PD, the number and intensity of intercellular communications were most significant (Figure 5B). Next, we were curious about which signaling pathways and ligand–receptor pairs (L-R pairs) change the cell communication network. We further compared the information flow for each signaling pathway between PD and CN, and found that some pathways such as the PSAP, VTN and SEMA4 pathways were turned off in PD, while the NRXN, NEGR, CNTN, NGL, EPHB, AGRN and CXCL pathways were turned on only in PD (Figure 5C). Moreover, we studied the detailed changes in the outgoing and incoming signaling across all pathways using pattern recognition analysis. Four pathways were specifically active in PD, including known nerve cell adhesion signals NRXN, NEGR, CNTN and NGL, suggesting that these pathways might critically contribute to disease progression. We also found that all PD turned on pathways maintaining outgoing and incoming patterns in ENDO_Rbfox1^High^ cells. In addition, four significant pathways (NRXN, NEGR, CNTN and NGL) exhibited the most prominent outgoing and incoming signaling patterns in AST1_Meg3^High^, AST_Luzp2^High^ and AST_Lsamp^High^ cells (Figure 5D). Corresponding to the signaling pathway, we also identified the PD up-regulated L-R pairs NRXN3–NLGN1, NRXN1–NLGN1 participating in the NRXN pathway and Negr1–Negr1 in the NEGR pathway, contributing to the communication among almost all AST1 and ENDO subclusters, especially autocrine and paracrine signaling between AST_Meg3^High^ and ENDO_Rbfox1^High^ cells (Figure S9).

In order to explore the communication network among D2-MSN, AST1 and ENDO subclusters, we conducted cell communication analysis on PD data alone. We found that D2-MSN communicated with almost all AST and ENDO subclusters, especially with AST_Meg3^High^ and ENDO_Rbfox1^High^ (Figure 5E, Figures S10A and S11). In addition, almost all AST subclusters contributed more receptors for D2-MSN, especially AST1_Meg3^High^, AST1_Lsamp^High^ and AST1_Luzp2^High^ cells (Figure 5E and Figure S11). The outgoing and incoming signals contributed equally and strongly between D2-MSN and ENDO_Rbfox1^High^, indicating that these two cell types were more closely interlinked (Figure 5E and Figure S11). In-depth exploration of the NRXN and NEGR signaling pathways indicated that these two signaling factors play a key role in the communication network between D2-MSN, ENDO_Rbfox1^High^ and AST1_Meg^High^. D2-MSN and ENDO_Rbfox1^High^ exhibited high expression of the sender, receiver, mediator and influencer, while almost all AST1 cells acted as influencers (Figure 5G and Figure S10D). Notably, we observed that the communication probabilities of NRXN3–NLGN1, NEGR1–NEGR1 and CNTN1–NRCAM interactions were more significant (*p*-value < 0.01) between these three cells (D2-MSN, ENDO_Rbfox1^High^ and AST_Meg3^High^) (Figure S10C), suggesting that MPTP induced huge cell communication network changes between these three PD-risk clusters by increasing NRXN3–NLGN1, NRXN1–NLGN1 and NEGR1–NEGR1 expression and enhancing the NRXN and NEGR signaling pathways. Our analysis suggested that the alteration in intercellular communications involving AST, ENDO and MSN cells might be a previously underestimated aspect of MPTP-induced PD pathogenesis, providing a basis for further exploration.

## 3. Discussion

In this study, we performed snRNA-seq combined with advanced bioinformatics analysis to explore the effects of MPTP on cell states in the mouse brain. We found PD-related cell population alterations, including AST1 and ENDO, and observed changes in their activation states. We also excavated some candidate TFs and genes that might be disease-related. In addition, we identified a PD-specific D2-MSN with significant changes in cellular status and gene expression. Finally, we observed enhanced cell-to-cell communication of these cells in PD. Our analysis of an MPTP-induced PD mouse brain provides a reference for understanding the cellular heterogeneity underlying disease pathogenesis.

MPTP has been shown to cause pooling of blood in the brain microvasculature and decrease the permeability of the BBB, and BBB dysfunction is involved in the course of PD. BBB is mainly composed of ENDO, pericytes and AST, and reactive gliosis is a common feature of AST during BBB destruction [60]. In our dataset, we identified PD-specific astrogliosis and found its activation status and the activation of TFs in disease. We observed that the AST1 subpopulations transition from a resting to an activated state with the transition from a healthy to a diseased state. It has been reported that AST is characterized by a stellate shape with multiple processes and ramifications, and becomes activated following brain injuries and degenerative diseases [12]. In addition, we found some potential PD marker genes associated with AST that are linked with cognitive impairment, and the highly expressed genes (*Luzp2* and *Lsamp*) of the activated subclusters in PD were distributed in the hippocampus in the Allen Brain Atlas. Although it has been demonstrated that AST can be activated in the striatum of PD [12], the hippocampus is also implicated in the cognitive dysfunction seen in some patients with PD. The lack of similar immunofluorescence experiments for verification is a deficiency of this paper, but we boldly speculated that PD-related AST cells might come from or be part of the hippocampus. Finally, we found that AST1 activated cell subpopulations in PD associated with the endocannabinoid trans-synaptic and glutamate receptor signaling pathway. The endocannabinoid system can modify dopamine transmission through glutamatergic synapses, and its signaling pathway is involved in the pathophysiological process of MPTP-inducted PD [61]. Evidence suggests that glutamate excitotoxicity may also play roles in the neurotoxicity of MPTP [11]. We also found heat-shock proteins (HSPs, Hspa1a) overexpressed in AST1 of PD, which is consistent with PD-specific microglia in the PD human midbrain [8]. HSPs have been shown to be protective towards the hypothesized mechanisms of MPTP toxicity [11]. HSPs, as specific molecules produced by AST, may be a promising neuroprotective strategy in neuropathology. Therefore, we speculate that our AST1-related genes will provide important clues for PD research.

TFs tightly control cell fate in neurodevelopment and have been implicated in neurodegenerative processes. We found that most of the PD-specific activated TFs were distributed in AST1, and then identified the TFs most linked with AST1 in the disease model: Dbx2 and Sox13. Studies have shown that Dbx2 encodes developing brain homeobox protein 2, highly expressed during neuronal development and regulating the differentiation of interneurons in the brain and spinal cord [62]. The widespread Dbx2 expression can have an effect on gross motoric function in fruit fly and mice [63]. In addition, Dbx2 has recently been shown to act as a TF regulating the maturation of cultured AST [64]. Similarly, the Sox gene family functions as important transcriptional regulators of glial development in the central nervous system [65]. Subsequently, we clarified that these TFs play inhibitory or rewarding roles in disease, which had not been reported in any previous studies, and our analysis provides potential target markers for the treatment of PD.

The BBB is characterized by the presence of tight junctions between ENDO cells and the expression of specific polarized transport systems, and some studies have shown alterations in ENDO tight junctions during PD development [12]. Our multi-channel analysis showed that MPTP-induced PD mice were closely related to ENDO cells. We simulated the activation trajectory of ENDO cells and detected the specific deletions of reactive ENDO in PD, especially in the subset of cells of Bsg^High^. *Bsg* plays a crucial role in angiogenesis, and its co-expressed genes tended to be enriched in gene terms of the extracellular matrix, cell adhesion and cell–cell interactions [66]. We hypothesized that the loss of ENDO cells in PD resulted in the destruction of tight junctions between cells, thus enhancing the permeability of BBB and promoting the entry of MPP^+^ toxins into the brain environment. In addition, we also explored some genes in ENDO cells that affect neurological diseases and BBB, which might be used as candidate markers for PD diagnosis. Among them, *Cxcl12* levels may be potential biomarkers of inflammation in PD patients, and *Slco1a5*, *Cldn5* and *Ly6a* genes are all associated with the BBB [67]. Furthermore, we found low expression of HSPs in PD-specific ENDO cells, contrary to AST1. The rise in HSPs level confers tolerance to energy deprivation, which is one explanation for the neurotoxic effects of MPTP, and our results also confirm this view [11].

Our snRNA-seq data also showed a trend towards increased cells of OLG and OPC in PD, but our trajectory analysis results did not observe a significant PD-risk association for OLG and OPC, which was consistent with the results of the latest genome-wide association studies [68]. This suggests that MPTP intake may not be the driving factor for the changes in OPC and OLG cells’ status. Namely, MPTP can induce more gene expression and cell state changes in AST1 and ENDO than in OLG and OPC.

Although neuronal cells should have been one of the focuses of our study, in-depth analysis was not carried out due to the lack of more accurate information to reveal their identity. However, we still found a PD-specific neuron and then decrypted its identity and status. We discovered a subcluster 14 with high expression of *Rarb*, *Pde7b*, *Rgs9* and *Gnal* that was unique to PD, which was defined as D2-MSN in the striatum. PD pathologies lead to the malfunction of the nigrostriatal dopamine pathway, where dopaminergic neurons release dopamine from axon terminals to the MSN in the dorsal striatum [2]. Although D2-MSN cells should also be detected in CN, our data separated D2-MSN to only contain PD samples. We observed that D2-MSN-linked genes were associated with morphine and cocaine addiction pathways. 4’-Methyl-alpha-pyrrolidinopropiophenone (MPPP) is related to morphine, piperidine and other drugs, while MPTP is an impurity in the production process of MPPP. The opioid system is involved in the reinforcing phenomenon induced by many drugs such as cannabinoids, cocaine and nicotine, and also alcohol. Imaging studies have shown that the opioid system is involved in pain processing, and also in addiction, neuropsychiatric manifestations, feeding and food disorders and, finally, movement disorders and levodopa-induced dyskinesias [69]. Dopaminergic neurons in the substantia nigra are well known as being selectively vulnerable to the MPP^+^ effects, so we speculated that our PD unique D2-MSN might be subjected to different levels of toxin attack and suffer huge changes in cell state and gene expression.

Most scRNA-seq studies of PD mainly focused on the cell-type-specific gene expression patterns. To our knowledge, there have been few studies characterizing the cell–cell communication with scRNA-seq data in PD research. At the most analytical stage of this work, we observed that the injection of MPTP reduced the quantity of communication among AST, ENDO, and D2-MSN cells but increased the intensity of interaction, which may be related to the energy conservation hypothesis of the PD mechanism [11]. The results of CellChat analysis not only further confirmed the accuracy of our identification of PD-specific cell types, but also revealed the synergistic communication relationship among these cells. We found signaling pathways of PD-specific cells, including NRXN, NEGR, CNTN and NGL, and their roles in PD have not been reported in the literature. NRXN and NLGN are trans-synaptic proteins involved in vascular biology, and the synaptic proteins of the NRXN family are involved in the vascular system through their interaction with a basic vascular cell [70]. Studies have found that NRXN–NLGN links synaptic function to cognitive disease [71], and the mutations in the NRXN-1 and CNTN4 genes have been reported to cause autism spectrum disorders (ASD) [72]. NEGR1 is a generic risk factor for multiple human diseases, including obesity, autism and depression [73]. It has been reported that NRXN and NLGN proteins are not suitable biomarkers for AD synapse pathology, but in our data their pathways were closely related to AST and ENDO in PD. We speculate that they may be potential markers for PD pathology and may be related to MPTP intake.

In summary, our study revealed several aspects of PD pathology caused by MPTP. Initially, we identified a disease-specific up-regulation of AST as well as the loss of ENDO cells, and systematically catalogued candidate target genes and TFs that might be associated with PD. In addition, we discovered a D2-MSN cell that exists only in MPTP-PD, which is an independent cell state initiated during MPTP induction. Finally, the cell–cell communication between PD-specific cells was investigated in detail, and identified the PD-related signaling factors and L-R pairs. Taken together, our work at least partially supports the changes in disease-specific cells and genes in MPTP in the mouse brain, and we hope that this study will provide a reference for the pathogenesis interpretation of PD.

## 4. Materials and Methods

### 4.1. Ethics Statement

The study was approved by the animal ethical and welfare committee of Zhongda Hospital Southeast University. All procedures were conducted following the guidelines of the animal ethical and welfare committee of SEU. All applicable institutional and/or national guidelines for the care and use of animals were followed.

### 4.2. Tissue Dissection and Nuclear Extraction

Eight-week-old male MPTP-induced Parkinson model mice (on a C57BL/6J background, MPTP-PD) and recommended control (C57BL/6J, CN) were purchased from the Shanghai Model Organisms Center, Inc, Shanghai, China. The animals were anesthetized with 500 mg/kg tribromoethanol (Sigma, Saint Louis, MO, USA) and were killed by cervical dislocation. After the animals were sacrificed, brain tissues (cerebral cortex, hippocampus, striatum and cerebellum) were isolated, quickly frozen in liquid nitrogen and stored in liquid nitrogen (n = 1). Four brain regions were pooled for nuclei isolated according to the ‘Nuclear Isolation by Single-cell RNA Sequencing’ protocol of 10X Genomics^®^. In brief, the tissue was lysed in a chilled lysis buffer (10 mM Tris-HCl, 10 mM NaCl, 3 mM MgCl2, 0.1% NP-40). Then, the suspension was filtered and nuclei were pelleted by centrifugation. Nuclei pellets were then washed in ‘nuclei wash and resuspension buffer’ (1× PBS, 1% BSA, 0.2 U/μL RNase inhibitor, 2 mM DTT), filtered and pelleted again. Cell count was then performed to calculate the concentration of nuclear suspension.

### 4.3. Library Construction and Sequencing

Sorted nuclei were processed using the 10× Chromium Next GEM Single Cell 3’ Kit v3.1 to generate the cDNA libraries. The quality of cDNA was assessed using the Agilent 2100 Bioanalyzer System. Sequencing was performed on Illumina NovaSeq 6000-S2.

### 4.4. Data Demultiplexing and Quality Control

We used Cell Ranger 5.0.1 (10 × Genomics) to process raw sequencing data, and the Seurat v4.0 was applied for downstream analysis. Before we started downstream analysis, we focused on four filtering metrics to guarantee the reliability of our data. (1) Genes detected in fewer than three cells were filtered to avoid cellular stochastic events; (2) nuclei with a percentage of expressed mitochondrial genes greater than 10% were removed to rule out apoptotic cells; (3) cells with UMI greater than 10,000 were removed to filter out the doublet-like cells; (4) cells with detected genes out of the range of 200–4000 were removed. After filtering cells and genes according to the metrics mentioned above, we further applied Doublet Finder V2.0 with default parameters to predict and remove potential doublets within each sample. As a result, there were 22,431 genes and 19,531 nuclei left for downstream analysis.

### 4.5. Clustering and Cell Annotation

After quality control, unsupervised clustering was performed using Seurat v3 [74] in a region-independent fashion. A series of preprocessing procedures including normalization, variance stabilization and scaling data, were performed in an R function ‘SCTransform’ based on regularized negative binomial regression. Then, we selected 3000 highly variable genes to integrate all sequencing libraries (including PD and CN) using ‘FindIntegrationAnchors’ and ‘IntegrateData’ functions, followed by the regression of technical noise. Principal component analysis (PCA) was performed using integrated output matrix, and principal component (PC) significance was calculated using the ‘JackStraw’ function. In this case, we chose the top 30 significant PCs for downstream cluster identification and visualization. Clusters were defined based on ‘FindClusters’ function with resolution equal to 0.8. After the primary clustering analysis, we found a high proportion of excitatory neurons with high gene expression similarity. Therefore, we applied 2-rotation cluster strategy. Briefly, after the first clustering analysis, we obtained major cell types, then we subclustered the excitatory neurons with resolution equal to 0.25 and merged the 2-rotation results as final cluster results. Uniform Manifold Approximation and Projection (UMAP) was used for the final dimension reduction and visualization.

Based on the cluster results, we next used ‘FindAllMarkers’ function with MAST algorithm, which was specially developed and applied to single cell data detecting differential expressed genes to identify marker genes for each cluster. We ranked the marker genes according to the p-value and log_2_ fold change (log_2_ FC) within each cluster and searched top genes in Cell Marker [75] and Panglao DB [76] databases to annotate cell types of clusters.

### 4.6. Differential Expressed Genes Analysis

Within each cluster, we detected differential expressed genes (DEGs) between PD and CN conditions by using ‘FindMarkers’ function. we used ‘MAST’ setting as well and controlled false-discovery rates (FDRs) using the Benjamini–Hochberg procedure. Then we set threshold q_adjust < 0.05, abs∣log_2_ FC∣ > 1 to filter DEGs and obtained PD up- and down-regulated genes compared to CN for each cluster. The DEGs functional enrichment analysis based on GO and KEGG was applied by an R package ClusterProfile [77] v3.18.4 using a hypergeometric test and corrected for multiple hypothesis by FDR. We used R package wordcloud2 to show the frequency of PD-risk genes detected in differentially expressed genes within each cluster; the bigger word size indicates the greater frequency.

### 4.7. Inference of Regulon, Quantify Cell-Type Specificity Score and Functional Validation

To predict gene regulatory networks using single nuclei gene expression data, we used pySCENIC [18] approach. There are three major steps of SCENIC to construct high-confidence gene regulatory networks. First of all, SCENIC calculates co-expression modules between TF and candidate target genes using GENIE3. Then RcisTarget is used to create regulon with only direct targets by identifying modules for which the regulator’s binding motif is significantly enriched across the target genes. Finally, AUCell scores each regulon’s active score in each cell and creates a binarized activity matrix between regulons and cells. Using this matrix, we can predict cell states without removing batches and identify cell-type specifically activated regulons.

For identifying cell-state-specific regulons, we adapted an entropy-based method to quantify cell-type specific score of each regulon. Firstly, we name the vector of ( $P_{1}^{R}$ ,…, $P_{n}^{R}$ ) as P^R^ to describe the distribution of regulon activity score, the vector of ( $P_{1}^{C}$ ,…, $P_{n}^{C}$ ) as P^C^, which can indicate whether a cell belongs to a specific cluster, n is the total cell numbers. Then we calculate the Jensen–Shannon Divergence (JSD) from P^R^ and P^C^

$$\mathrm{JSD}\left(P^{R},P^{C}\right)=H\left(\frac{P^{R}+P^{C}}{2}\right)-\frac{H\left(P^{R}\right)+H\left(P^{C}\right)}{2}$$

where $H\left(P\right)=-\sum p_{i}\mathrm{loa}\left(p_{i}\right)$ .

Finally, the regulon specificity score (RSS) is calculated as this

$$\mathrm{RSS}\left(R,C\right)=1-\sqrt{\mathrm{JSD}\left(P^{R},P^{C}\right)}$$

Therefore, we know the range of RSS is (0, 1), and if the regulon activity is highly different among clusters, the RSS approaches 1, otherwise, if there is no difference of regulon activity among clusters, the RSS will be equal to 0.

To further validate whether our predicted regulons are functionally related to their associated cell types or PD condition is specifically activated, we employed an online tool SEEK [78]. SEEK provides the gene co-expression search function for lots of mouse database from the GEO (Gene Expression Omnibus), so we can detect whether the genes within the same regulon are co-expressed and which kinds of papers’ data had also reported the similar co-expression module. If genes within regulon are significantly co-expressed in many datasets related to Parkinson disease or some certain cell types, it could be further evidence that the regulon is reported to be highly related to Parkinson’s disease in a certain cell type.

### 4.8. Trajectory Analysis Using Monocle3

To obtain cellular state changes between PD and CN samples within AST1, ENDO, OLG, OPC and part of the excitatory neurons, we reconstructed the cellular states’ trajectories using the standard Monocle3 [26] workflow. Firstly, we subdivided certain clusters, used the filtered raw counts as input to integrate PD and CN cells and normalized factor size. The sample effect was removed using the Mutual Nearest Neighbor method with parameter ‘alignment_k = 20’. The reduce_dimension function was used for dimensionality reduction, and the Louvain method was used for clustering with a resolution of 0.01. Then, the trajectory inference used the learn_graph function with default parameters. Finally, pseudotime ordering was performed by rooting the trajectory manually based on the shape of trajectory and background knowledge.

The most important step is to identify trajectory-dependent genes that may influence the PD and CN cell states, slightly changing them within each cluster. We first calculated subcluster marker genes using ‘topmarkers’ function then using ‘graphtest’ function, which uses the spatial correlation analysis Moran’s I approach to identify highly variable genes associated with the trajectory. Thus, the trajectory-dependent genes were defined by intersection of subcluster markers and trajectory-associated highly variable genes.

### 4.9. Cell–Cell Communication Analysis

To further investigate the intercellular communication changes induced by MPTP, we used R software CellChat [79] v1.4.0 to calculate communication networks between subclusters of AST1, ENDO and D2-MSN. We predicted the communication network including signaling pathway and ligand–receptor (L-R) pairs information in PD and CN samples separately and then compared the network difference between these conditions. The interaction number and strength are two key factors, so we used ‘compareInteractions’ function to obtain whole network interaction number and strength differences. Then, for the conserved signaling pathways, we ranked these pathways according to their Euclidean distance in the shared two dimensions space. The top pathways indicated more difference between PD and CN. We also compared each signaling pathway’s information flow, which is the sum of communication probability among all cell pairs, to identify different pathway states including turn off/on, decrease and increase in one condition compared to the other. Finally, we zoomed in to the L-R pairs level, and calculated dysfunctional L-R pairs by using differential expression analysis with ‘identifyOverExpressedGenes’ and ‘netMappingDEG’ functions. The up-regulated and down-regulated L-R pairs can be detected. All the plot functions are from the CellChat package.

## Data Availability

The data that support the findings of this study have been deposited in the Gene Expression Omnibus (GEO) with accession number GSE205367.

## Supplementary Materials

The supporting information can be downloaded at: https://www.mdpi.com/1422-0067/23/18/10774/s1.
