# Supplementary material for: Defining Specific Cell States of MPTP-Induced Parkinson’s Disease by Single-Nucleus RNA Sequencing
Source: Int J Mol Sci. 2022 Sep 15;23(18):10774. doi: 10.3390/ijms231810774 (PMC9504791; doi:10.3390/ijms231810774)
Supplement: Supplementary file 1 [file ijms-23-10774-s001.zip › Supplementary Materials-Figures.pdf]

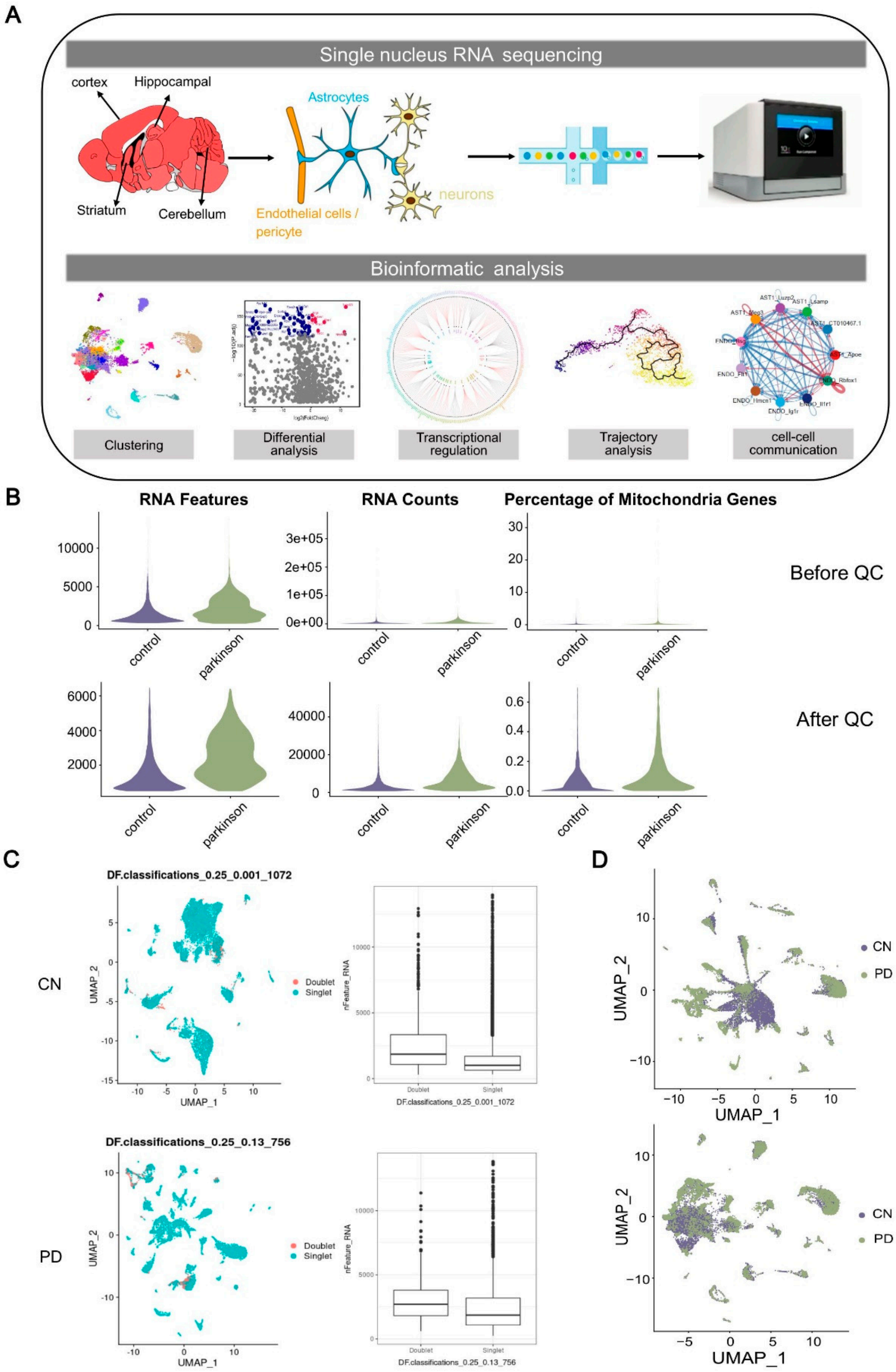

Figure S1: snRNA-seq quality control metrics and similarity. (A) Schematic representation of the samples used in this study, sequencing experiments and downstream bioinformatic analyses. (B) Information of samples before (top) and after (bottom) quality control. (C) Potential doublets were predicted and removed by the Doublet Finder V2.0. (D) 2D UMAP embedding of single nuclei RNA profiles before (top) and after (bottom) removing batch effect.

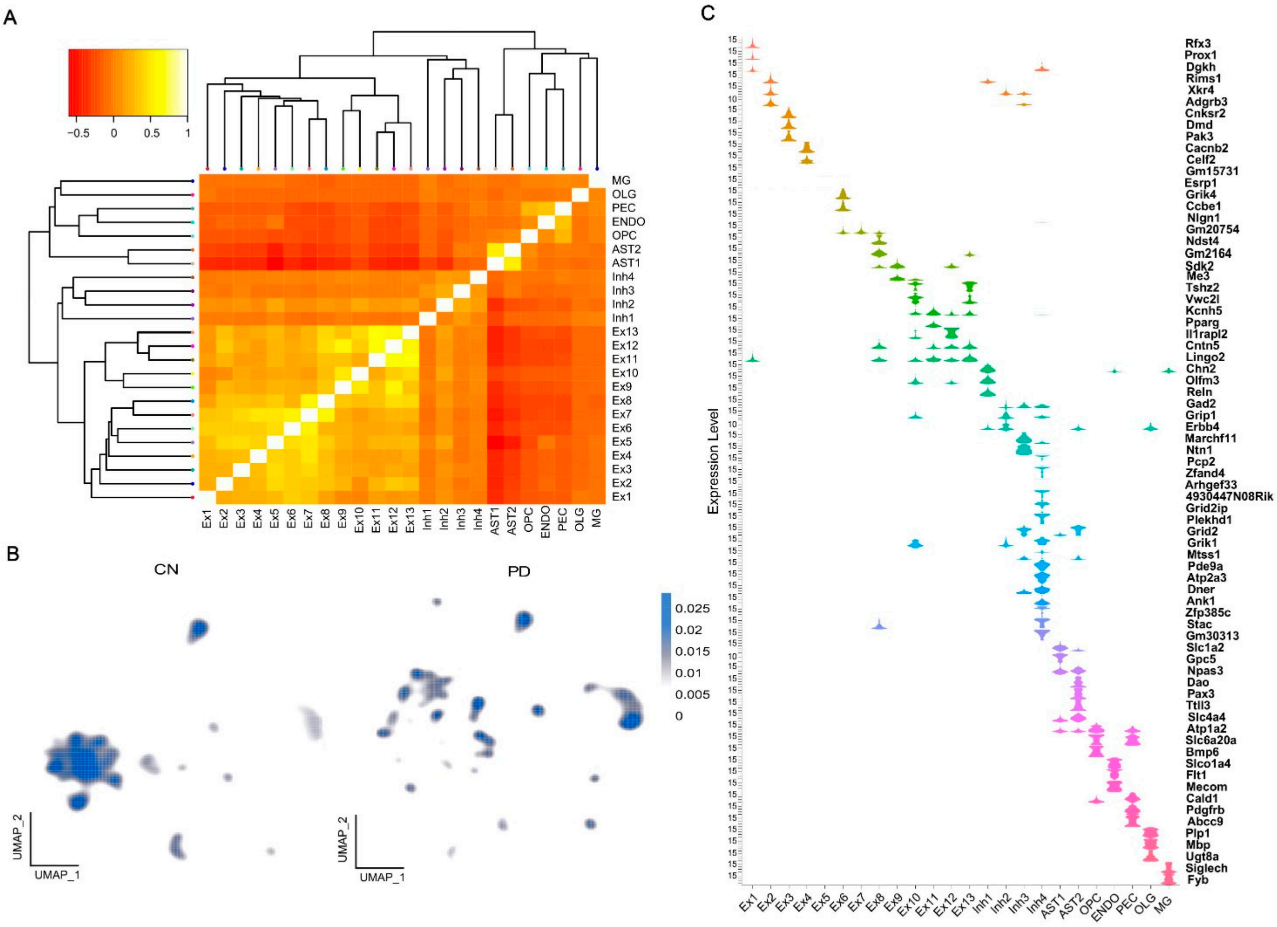

Figure S2: Cell type marker genes and assignments. (A) Confusion matrix results of the machine learning cross-validation approach to validate the cell type definition. (B) Expression distribution of cell-type marker genes. (C) 2D cell density UMAP embeddings for PD (right) and CN (left).

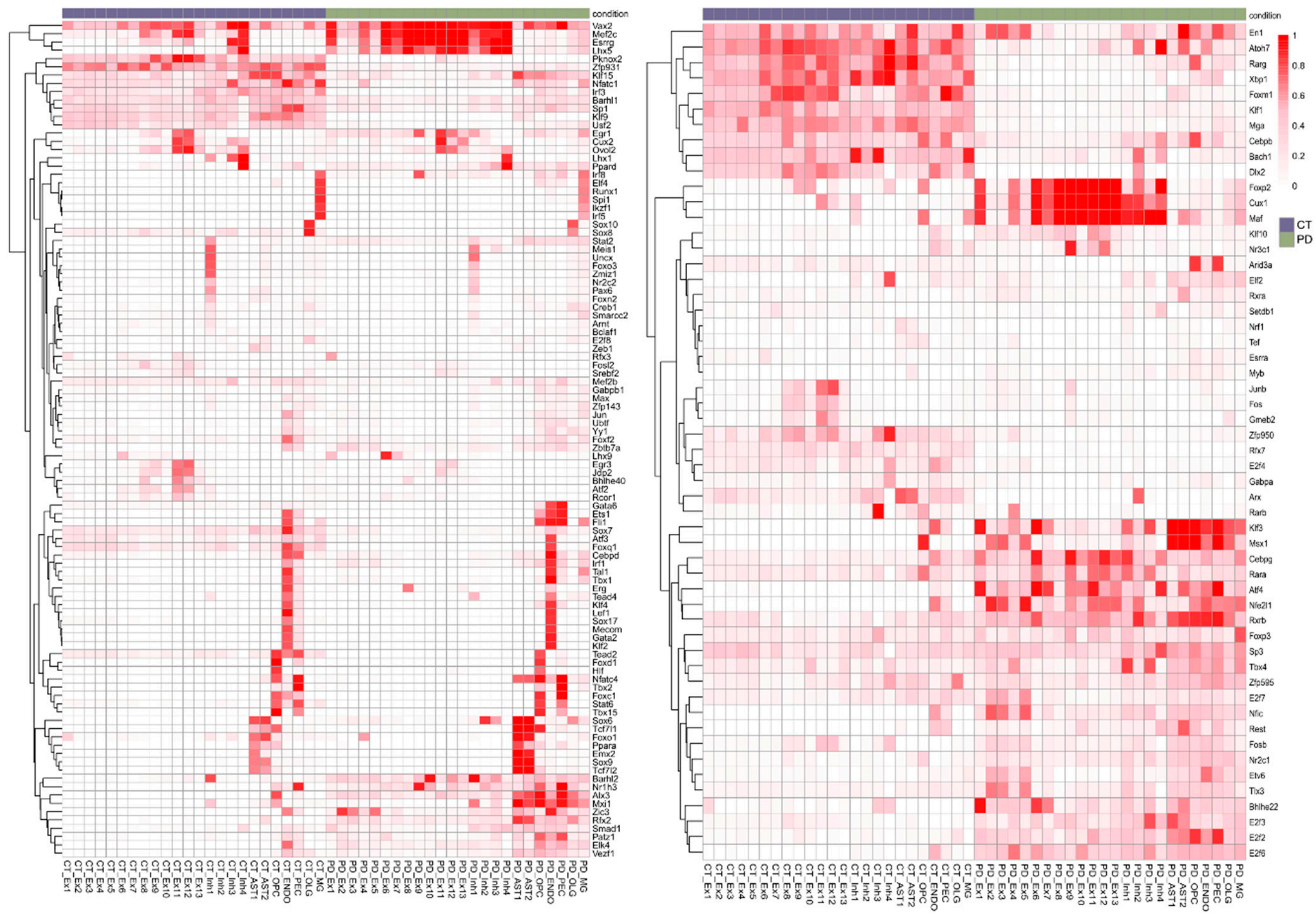

Figure S3: Heatmap of 155 TFs co-activated by PD and CN. There were 101 conservative TFs with similar activation status in PD and CN (left), and 54 TFs with specificity (right).

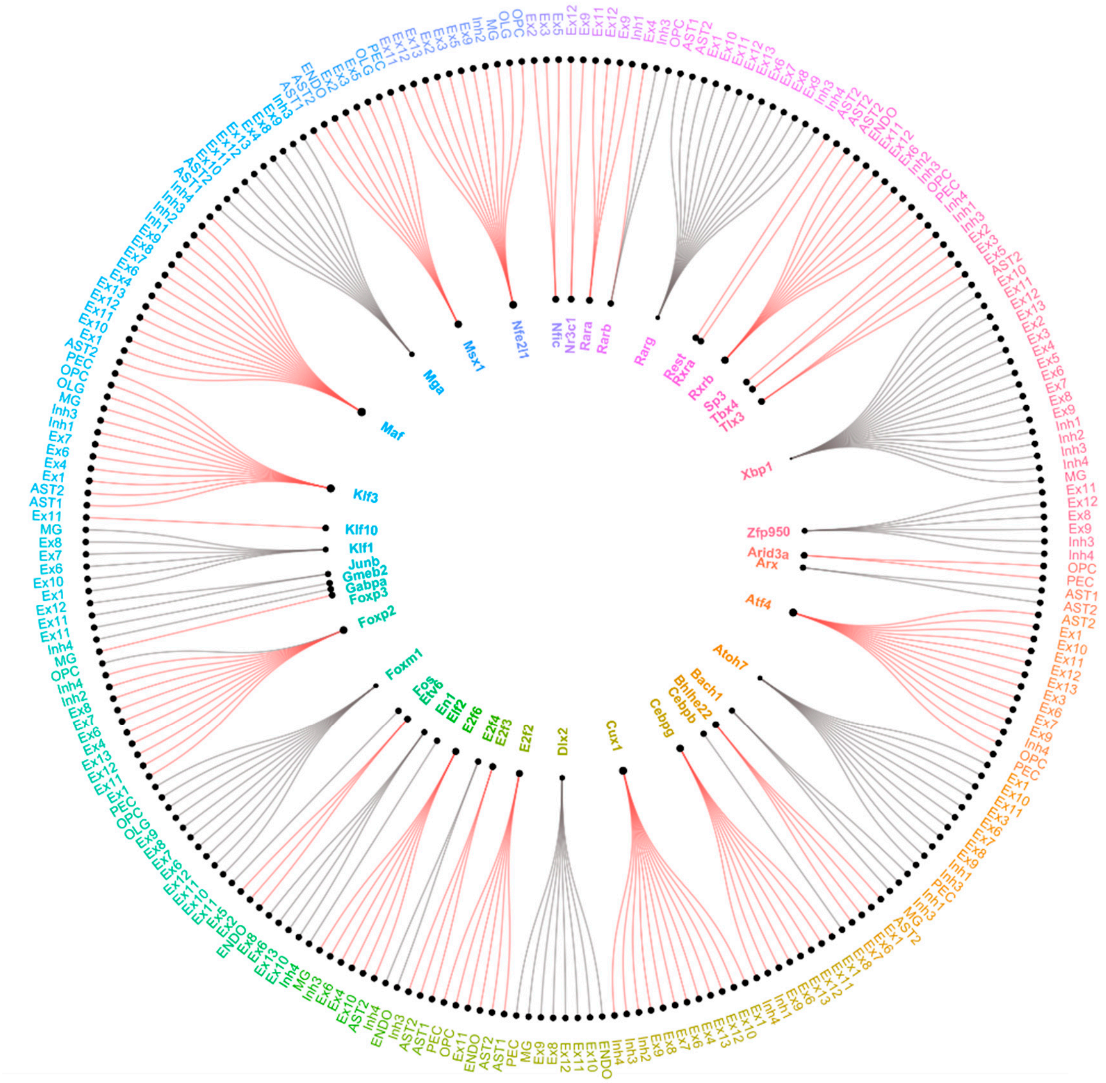

Figure S4: Cirle plot of 54 differentially activated TFs and their corresponding cell types in PD and CN (abs | pct (PD) – pct (CN) | >= 0.5). The inner ring is TFs, and the outer ring is a qualified cell types; The red and grey lines indicate activation in PD and CN respectively, and the size of the inner ring points indicates the number of differences.

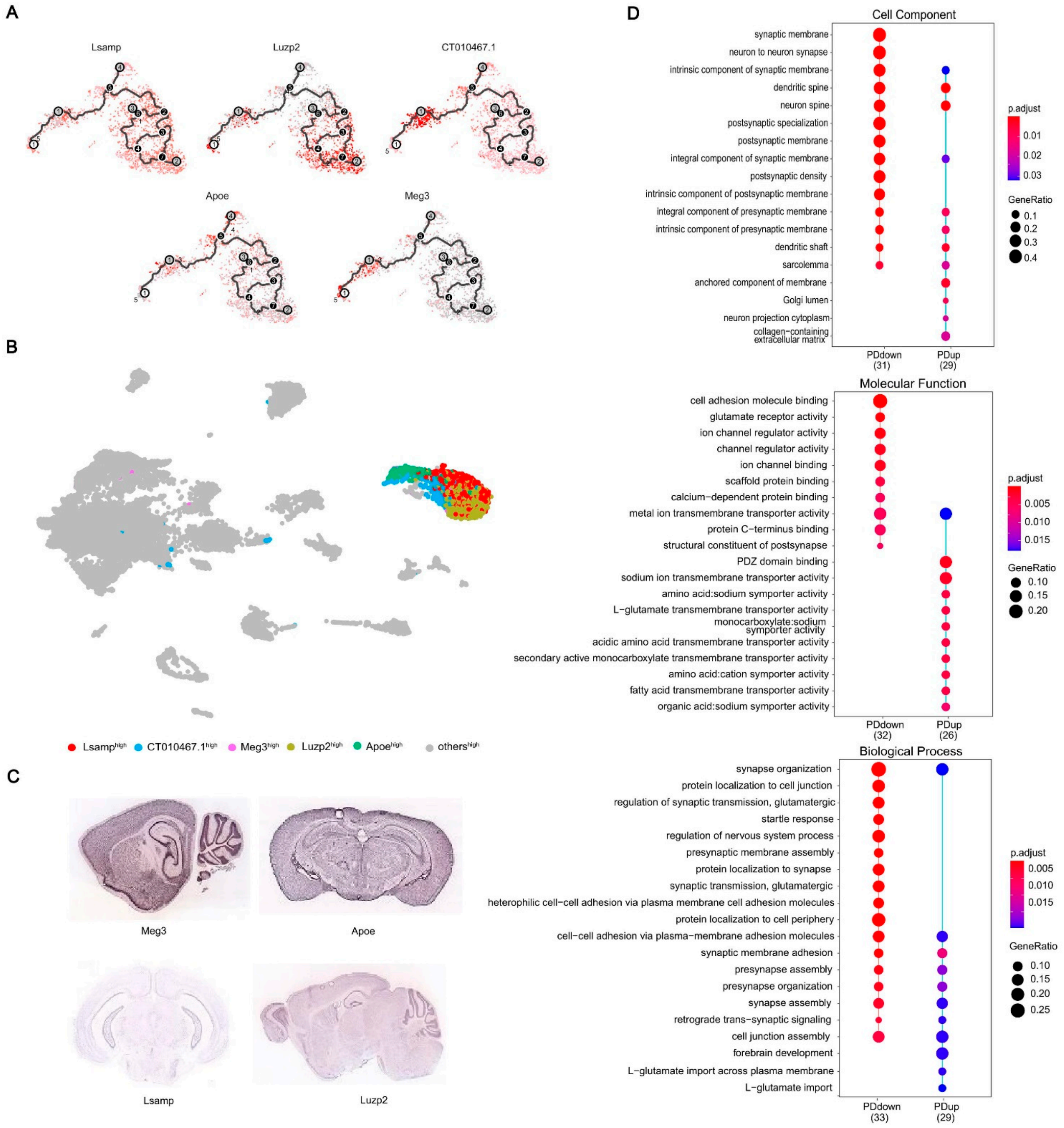

Figure S5: (A) The expression of marker genes along the AST1 subclusters. (B) UMAP embedding of five AST1 subclusters. (C) Distribution of AST1 subclusters marker genes in Allen Brain Atlas. (D) GO analysis of all AST1 subcluster DEGs. The color of the point corresponds to the value of p_adjust, and the size represents the proportion of the number of differential genes in the total number of differential genes under the GO term.

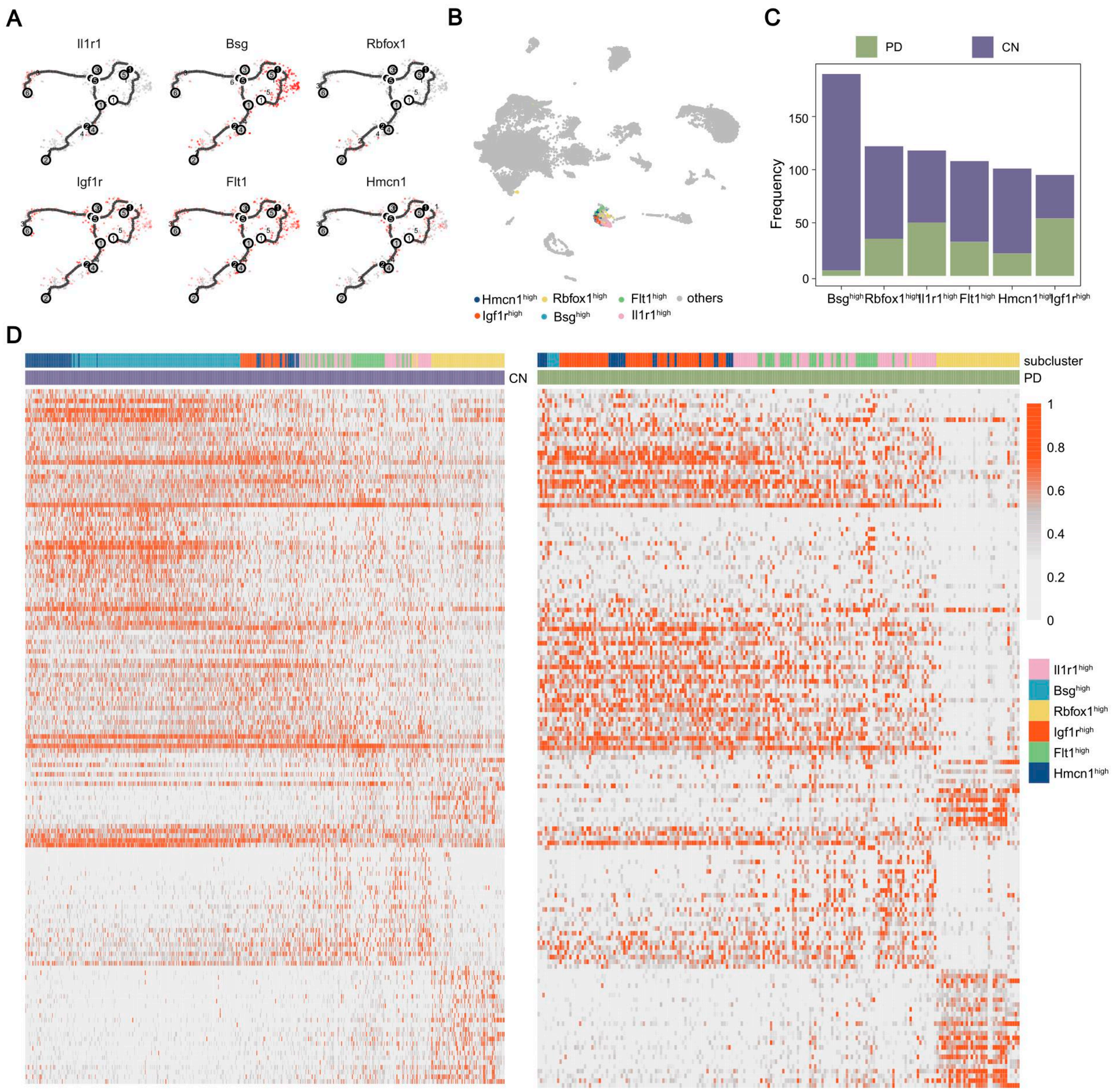

Figure S6: (A) The expression of marker genes along the ENDO subclusters. (B) UMAP embedding of ENDO subclusters. (C) the proportion of ENDO subclusters in PD and CN. (D) Trajectory dependent gene of each cell subcluster gene in PD (right) and CN (left).

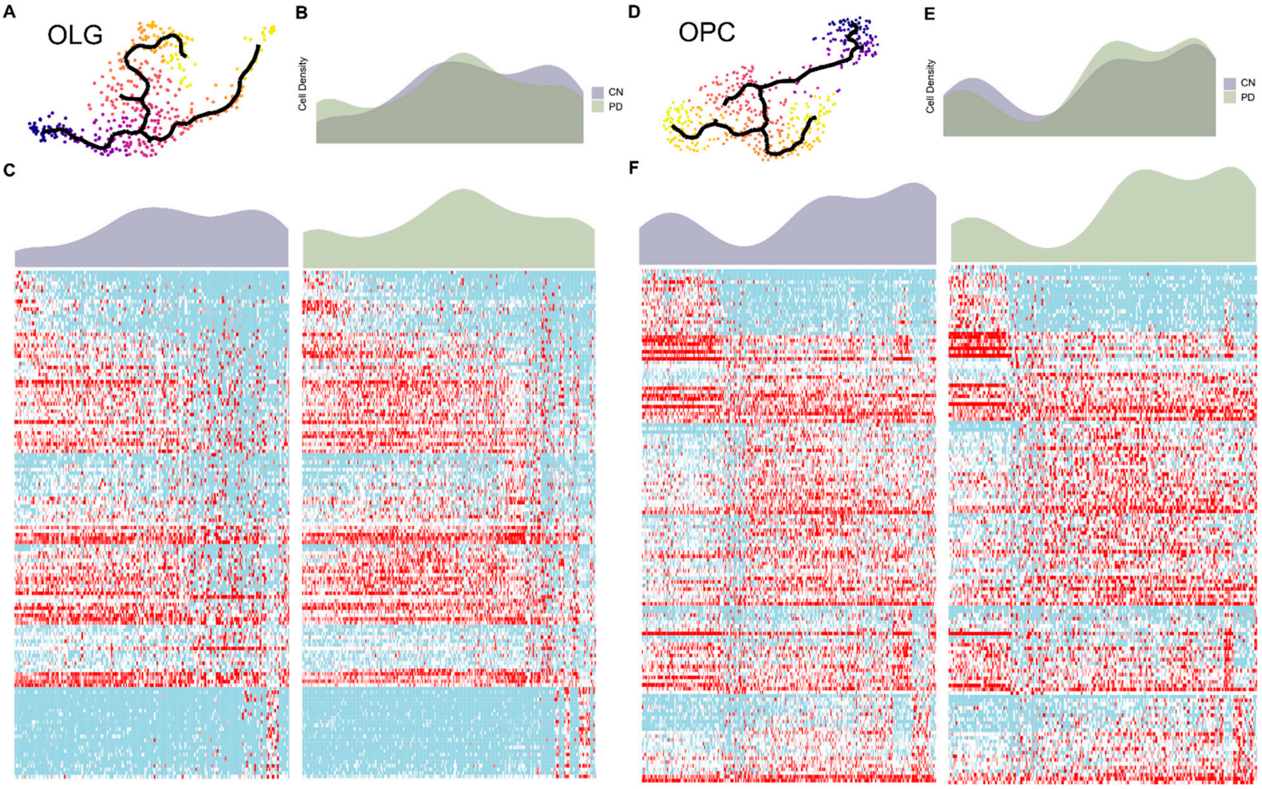

Figure S7: Trajectory reconstruction reveals oligodendrocytes and oligodendrocyte precursor cells differential activation in PD. (A, D) OLG and OPC trajectory reconstruction and pseudotime representation of subclusters. (B, E) differential cell-density distribution over pseudotime in CN and PD, separately. (C, F) Trajectory dependent gene of each cell subcluster gene in OLG (C) and OPC (F).

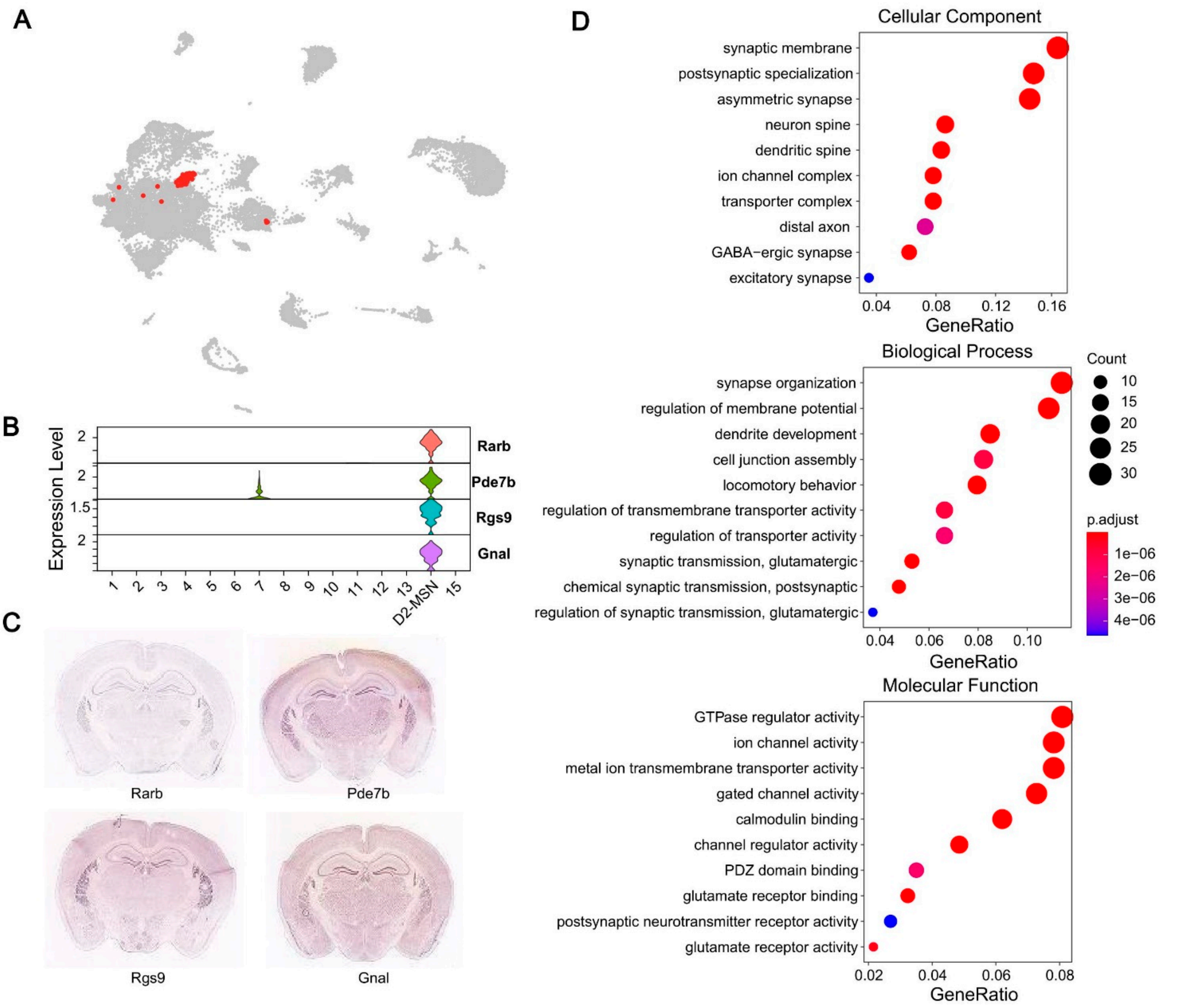

Figure S8: (A) UMAP embedding of subclusters 14, which overlaps with EX4. (B, C) The top four highly expressed marker genes of subclusters 14 in the ISH of Allen Brain Atlas. These genes are distributed in the striatum (STR). (D) GO analysis of all marker gene expression in subcluster 14. The color of the point corresponds to the value of p_adjust, and the size represents the number of differential genes under GO terms.

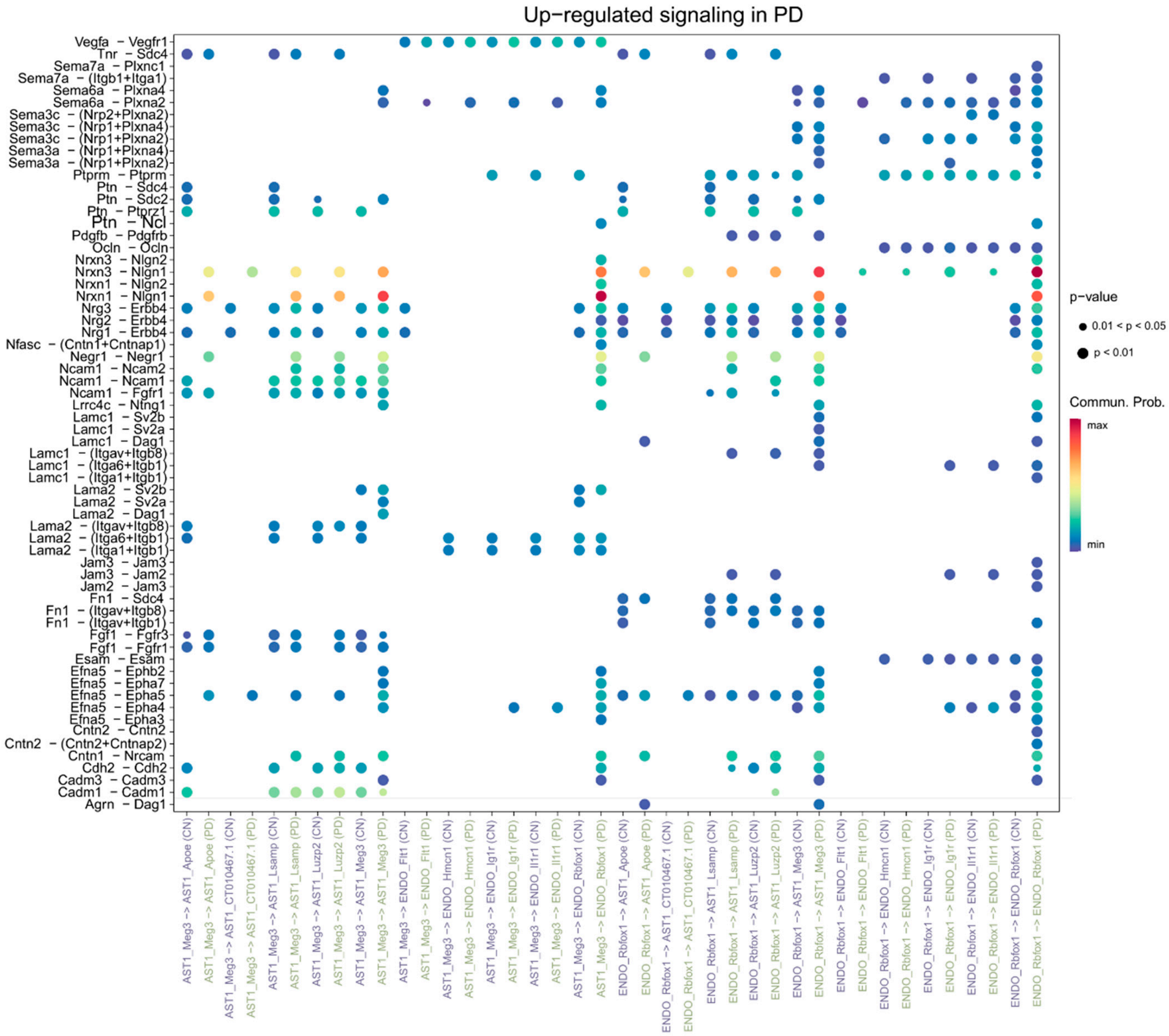

Figure S9: Up-regulated Ligand-receptor pairs that contribute to the signaling of PD-specific cells. The dot color and size represent the calculated communication probability and p-values.

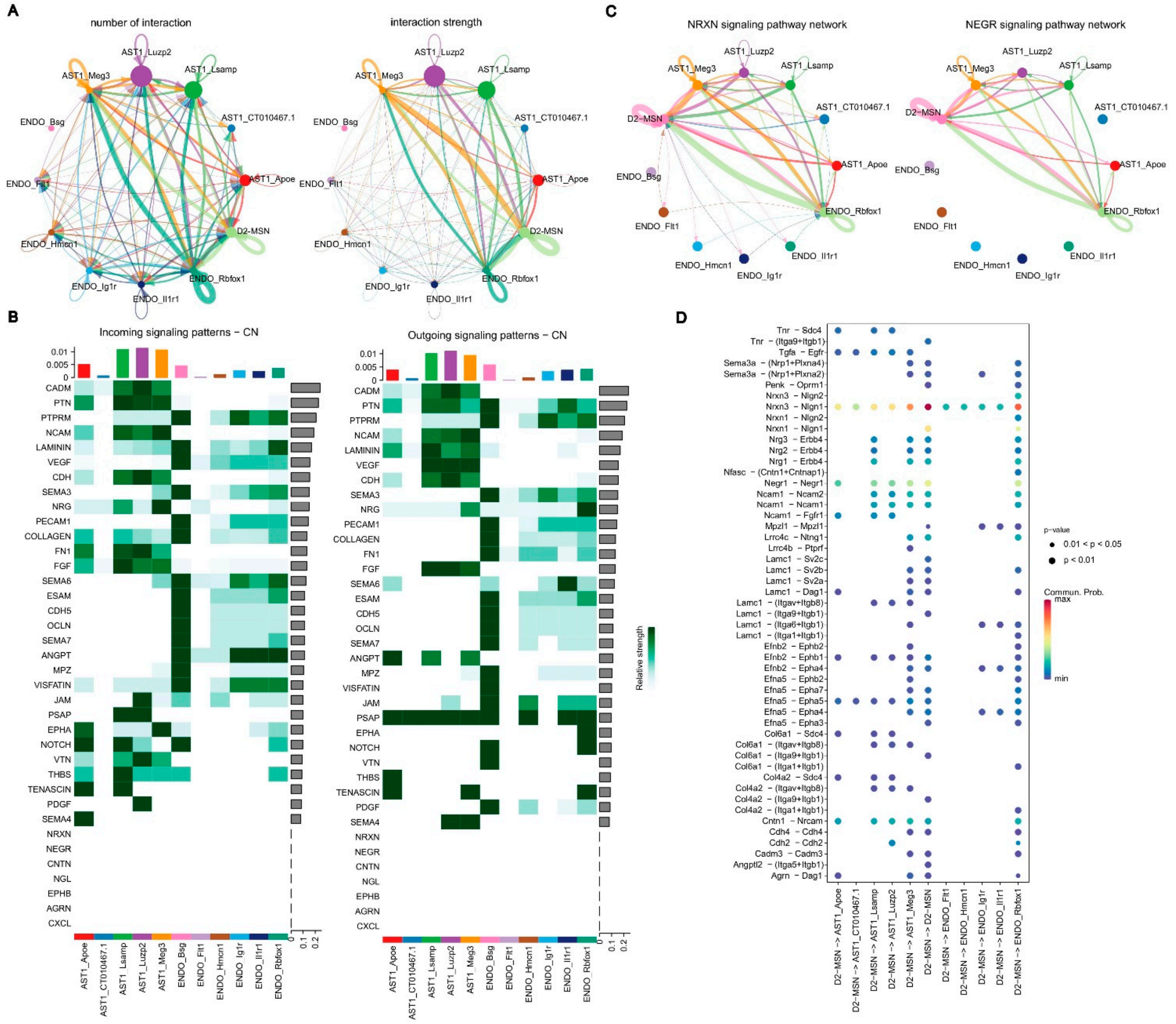

Figure S10: (A) Circle plot of the number and interaction strength of ligand-receptor pairs between PD-specific cells. (B) Heatmaps of the outgoing and incoming signaling patterns of AST1 and ENDO subclusters in CN. (C) Comparison of the significant ligand-receptor pairs between PD-specific cells, which contribute to the signaling from D2-MSN to AST1 and ENDO subpopulations. Dot color reflects communication probabilities and dot size represents computed p-values. (D) The inferred NRXN and NEGR signaling networks. Circle sizes are proportional to the number of cells in each cell group and edge width represents the communication probability.

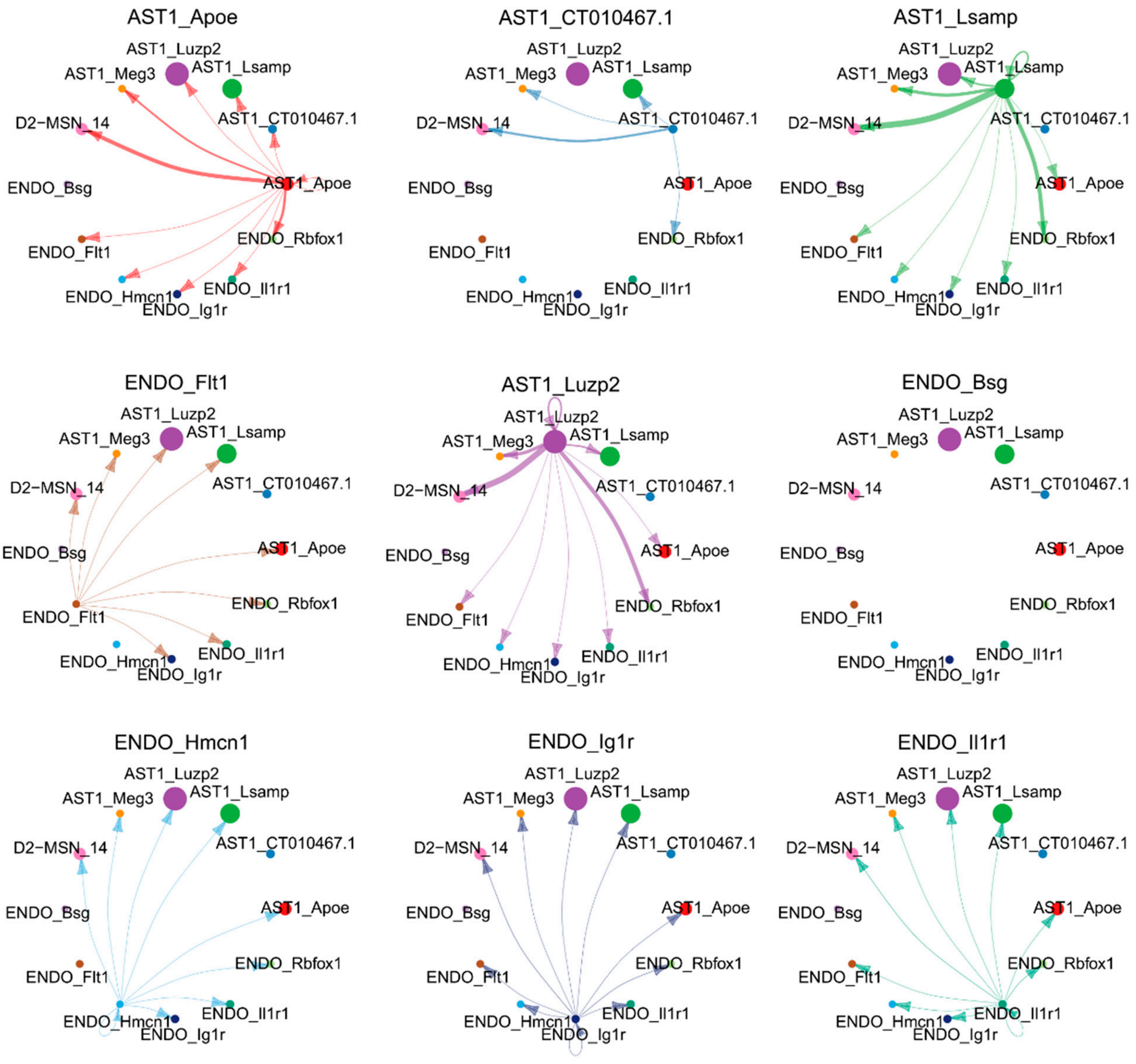

Figure S11: Circle plots of the interaction strength among D2-MSN, AST1 and ENDO subclusters in PD.
